# mRNA‐Engineered CD5‐CAR‐γδT^CD5‐^ Cells for the Immunotherapy of T‐Cell Acute Lymphoblastic Leukemia

**DOI:** 10.1002/advs.202400024

**Published:** 2024-07-16

**Authors:** Zhixiong Zhu, Hexian Li, Qizhong Lu, Zongliang Zhang, Jia Li, Zeng Wang, Nian Yang, Zhengyu Yu, Chen Yang, Yongdong Chen, Huaqing Lu, Wei Wang, Ting Niu, Chunlai Nie, Aiping Tong

**Affiliations:** ^1^ State Key Laboratory of Biotherapy and Cancer Center Collaborative Innovation Center of Biotherapy West China Hospital Sichuan University Chengdu 610041 China; ^2^ Department of Hematology State Key Laboratory of Biotherapy and Cancer Center West China Hospital Sichuan University Chengdu 610041 China; ^3^ Frontiers Medical Center Tianfu Jincheng Laboratory Chengdu 610212 China

**Keywords:** CAR‐T, IVT‐mRNA, T‐ALL, γδT cells

## Abstract

Clinical trials of Chimeric Antigen Receptor T‐cell (CAR‐T) therapy have demonstrated remarkable success in treating both solid tumors and hematological malignancies. Nanobodies (Nbs) have emerged as promising antigen‐targeting domains for CARs, owing to their high specificity, robust stability, and strong affinity, leading to significant advancements in the field of Nb‐CAR‐T. In the realm of T‐cell acute lymphoblastic leukemia (T‐ALL) targets, CD5 stands out as a potentially excellent candidate for T‐cell‐based CAR therapy, due to its distinct expression on the surface of malignant T‐ALL cells. To mitigate graft‐versus‐host disease associated with allogeneic CAR‐T, γδT cells are selected and stimulated from peripheral blood mononuclear cells, and γδT cells are engineered via CRISPR/Cas9 to eliminate fratricide, enabling the creation of fratricide‐resistant CAR‐γδT^CD5−^ cells. In vitro transcribed (IVT) mRNA is used to construct CAR‐T, presenting a safer, faster, and cost‐effective method compared to traditional viral vector approaches. In this study, a CD5‐VHH library is constructed, and specific CD5‐nanobodies are screened for subsequent use in CD5‐CAR‐γδT^CD5−^ therapy. IVT‐mRNA‐CD5‐CAR‐γδT^CD5−^ cells exhibited favorable functional characteristics and demonstrated antitumor efficacy against malignant T cell lines, underlining the potential for advancing mRNA‐CD5‐CAR‐γδT^CD5−^ therapy.

## Graphical abstract

A VHH library is successfully constructed, and the CD5‐27 Nb is selected for the CAR construct. γδT cells are amplified from PBMCs of healthy donors through IPP and IL‐2 stimulation. The CRISPR/Cas9 system is used to knock out CD5 in γδT cells to avoid fratricide. The IVT‐CAR‐mRNA‐engineered CD5‐CAR‐γδT^CD5‐^ exhibits efficient antitumor efficacy against malignant T‐cell lines.

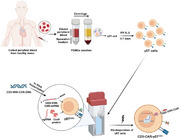

## 1 Introduction

Chimeric antigen receptor T‐cells (CAR‐T) are T lymphocytes that have been genetically engineered to express synthetic CAR molecules on their surfaces.^[^ ^1^ ^]^ These molecules are composed of an extracellular domain, specifically designed to recognize and bind to antigens on the surface of target cells.^[^ ^2^ ^]^ Nanobodies (Nbs) have been developed as antigen‐targeting domains for CARs, chosen for their high specificity, strong stability, and top affinity, leading to significant advancements in CAR‐T therapy.^[^ ^3^, ^4^ ^]^

CAR‐T therapy has achieved remarkable success in clinical trials.^[^ ^5^ ^]^ Several CAR‐T cell products have received regulatory approval, and ongoing research is expanding the application of this therapy to solid tumors and other hematological malignancies.^[^ ^6^ ^]^ Among the hematological malignancies, T cell acute lymphoblastic leukemia (T‐ALL) is an aggressive malignant neoplasm arising from the transformation of T cell progenitors,^[^ ^7^ ^]^ presents unique challenges, notably the scarcity of distinct target antigens for effective discrimination of leukemic from normal T cells.^[^ ^8^ ^]^ Previous studies have shown that CD5 is a potentially good target for CAR, as it is one of the markers expressed on malignant T cells in T‐ALL.^[^ ^9^ ^]^ Additionally, CD5 is not expressed on hematopoietic stem cells, minimizing the risk of off‐tumor effects.^[^ ^10^ ^]^ Moreover, preclinical studies have shown that CD5‐CAR‐T cells preferentially target malignant T cells while sparing the normal T population.^[^ ^11^ ^]^ Despite its promise, the innate expression of CD5 on CAR‐T cells induces fratricide, diminishing therapeutic efficacy.^[^ ^12^ ^]^ In that case, leveraging CRISPR‐Cas9 technology to knock out the CD5 gene in CAR‐T cells presents a pioneering approach, circumventing fratricide and bolstering the effectiveness of CD5‐targeted CAR‐T therapy for T‐ALL.^[^ ^13^, ^14^ ^]^

Simultaneously, T‐ALL, as a malignant hematological neoplasm originating from T‐cells, presents a formidable obstacle in harvesting patient‐derived normal T‐cells for CAR‐T therapy.^[^ ^15^ ^]^ In that case, CAR‐γδT cell therapy is in the spotlight and expected to break the plight. As each individual has their own unique set of human leukocyte antigen (HLA) molecules, the αβT cell immune systems cannot easily be transferred between individuals.^[^ ^16^ ^]^ In contrast, γδT cells do not rely on the recognition of classic HLA molecules, and sensing of infection or cancer depends on more ubiquitous changes observed across many individuals most of the time.^[^ ^17^ ^]^ Thus, γδT cells can be collected from healthy donors, and have the capability to achieve allogeneic transplantation, mitigating severe graft‐versus‐host disease.^[^ ^16^, ^18^ ^]^ Preliminary studies indicate that CAR‐modified γδT cells exhibit cytotoxic activity against various malignancies, including hematologic and solid tumors.^[^ ^19^ ^]^ However, challenges such as optimizing the CAR design for γδT cells, enhancing in vivo expansion and persistence, and mitigating potential off‐target effects are active areas of investigation.^[^ ^20^ ^]^

Traditionally, T cells are engineered through ex vivo transfection via viral vectors carrying CAR gene sequences and subsequently administered back to the patients to treat related diseases.^[^ ^1^, ^21^ ^]^ However, this procedure involves several aspects that can be optimized, such as the complexity of the CAR‐T cell preparation process and the associated risks of viral vector integration into the cell genome, potentially inducing tumor formation.^[^ ^22^, ^23^ ^]^ Therefore, in vitro transcribed mRNA CAR‐T (IVT mRNA CAR‐T) therapy is an emerging safe, rapid, and cost‐effective alternative to overcome these challenges, offering the advantage of circumventing long‐term adverse effects.^[^ ^24^ ^]^ Due to the labile nature of mRNA, IVT mRNA CAR‐T reduces the side effects associated with on‐target, off‐tumor toxicity.^[^ ^25^ ^]^ Besides, in vitro and in vivo studies have demonstrated the therapeutic ability of mRNA‐engineered T cells in solid tumors, including melanoma, neuroblastoma, and ovarian cancer.^[^ ^26^ ^]^ Moreover, in the clinical application, this approach provides better controllability over dosages and efficacy, holding significant potential for future developments.^[^^26]^

Despite the promising advances, the effectiveness of IVT mRNA CAR‐γδT therapy in treating T‐ALL awaits further validation. In our research, we explored the application of Nb to construct CD5 CAR and developed CD5‐CAR‐γδT cells. γδT cells were collected from healthy donors and engineered by CRISPR/Cas9 ribonucleoprotein (RNP) and electroporation with CD5‐VHH‐CAR mRNA to construct CD5‐CAR‐γδT^CD5−^. Preliminary findings affirm the desirable functional attributes of mRNA‐CD5‐CAR‐γδT^CD5−^ cells, underscoring the imperative for continued exploration into this innovative therapy.

## 2 Results and Discussion

### 2.1 VHH Library Construction and Specific Nanobodies Screening

**Figure 1:**
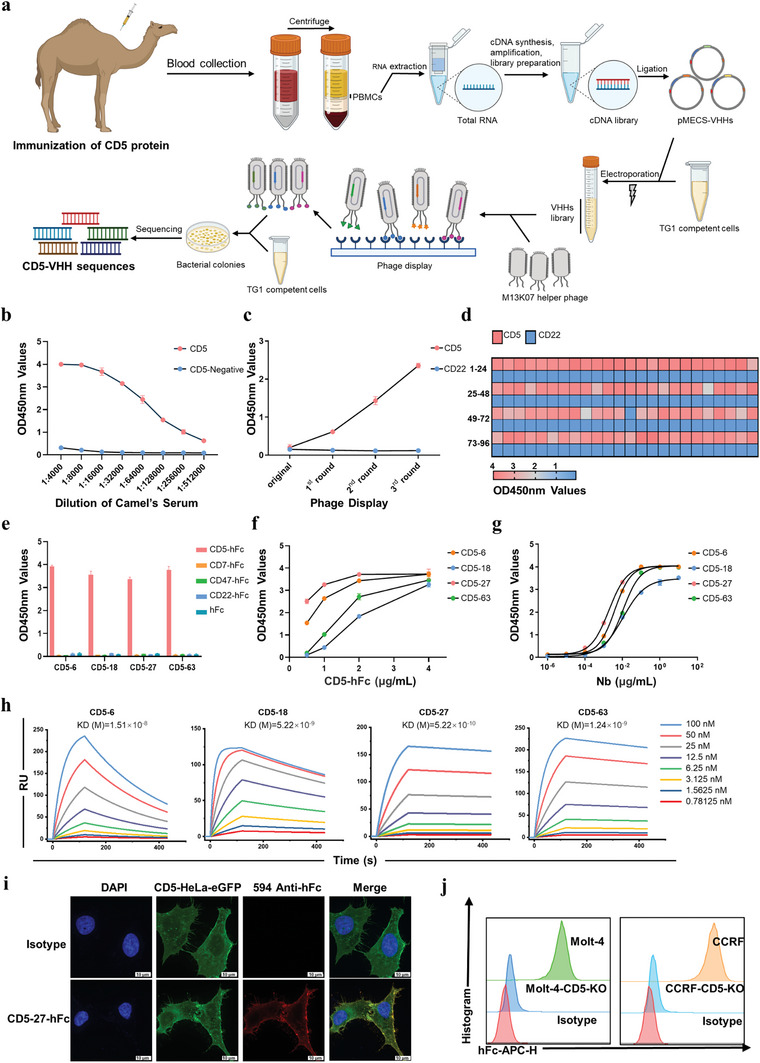
CD5‐VHH library construction, specific nanobodies screening, and characterization of the CD5‐Nbs. a) Schematic presentation of nanobody screening. b) Immunological valence analysis. c) Enrichment analysis of phage particles against CD5 protein‐specific nanobodies using polyclonal indirect ELISA. d) Identification of recombinant VHH‐gpIII proteins from the 96 clones specifically binding with CD5 protein. e) Analysis of the specific binding ability of recombinant monovalent nanobodies using indirect ELISA. f, g) Measurement of the binding ability of recombinant monovalent nanobodies using indirect ELISA. h) Real‐time binding profile of recombinant monovalent nanobodies with CD5 by SPR. i) Immunofluorescence for verifying the binding ability of CD5‐27‐Nb with CD5‐HeLa by CLSM. The scale bar represents 10 µm. j) Analysis of the binding ability of CD5‐27‐Nb with CCRF‐CEM and Molt‐4 by flow cytometry. The image of the schematic presentation of screening nanobodies is created with BioRender.com. ELISA, enzyme‐linked immunosorbent assay.

A healthy 3‐year‐old camel was immunized with high‐purity recombinant CD5 extracellular domain protein through the subcutaneous route following previously reported procedures.^[^ ^27^, ^28^ ^]^ After the third immunization, immunological valence was measured (**Figure** **1**b), and peripheral blood lymphocytes were isolated from 200 mL fresh blood samples by density gradient separation (Figure 1a). Total RNA of peripheral blood mononuclear cells (PBMCs) was extracted and reverse transcribed to cDNA using the SuperScript III First‐Strand Synthesis System (Thermo Fisher, USA). VHH genes were amplified and ligated into the pMECS phage vector and transformed into E. coli TG1 competent cells (Figure 1a). Three consecutive rounds of bio‐screening were performed to obtain specific nanobodies, and the monitoring of the screening process was conducted using polyclonal phage ELISA (Figure 1c). The positive VHHs were identified using monoclonal phage ELISA, and clones were randomly selected for sequencing to analyze the library's diversity (Figure 1d). This rigorous selection process culminated in the identification of 4 distinct nanobodies targeting CD5, based on the unique amino acid compositions of their complementarity‐determining regions.

### 2.2 Expression, Purification and Characterization of the CD5‐Nbs

The expression of CD5‐Nbs (CD5‐6, CD5‐18, CD5‐27, CD5‐63) was performed by HEK293T cells. Then, the specific binding of CD5‐Nbs with CD5 protein was assessed (Figure 1e). CD5‐27‐Nb was selected for its superior affinity and enhanced antitumor efficacy, as evidenced by ELISA (Figure 1f,g), surface plasmon resonance (SPR) (Figure 1h), and the real‐time cytotoxicity assays (RTCA, *xCELLigence*) system (Figure S1, Supporting Information). As shown in Figure 1i, CD5‐27‐Nb demonstrated the ability to bind to HeLa cells overexpressing CD5 (CD5‐HeLa) as observed through a confocal laser scanning microscope (CLSM). Besides, flow cytometry was performed to demonstrate that CD5‐27‐Nb efficiently binds to MOLT‐4 and CCRF‐CEM (Figure 1j), which are T‐cell malignancy cell lines that naturally express CD5.^[^ ^29^ ^]^ These findings collectively underscore the potential of CD5‐27‐Nb as a targeted therapeutic agent, warranting further exploration for its application in cancer immunotherapy.

### 2.3 Effective Disruption of the CD5 Gene in T Cells Prevents Fratricide Without Compromising T Cell Function

**Figure 2:**
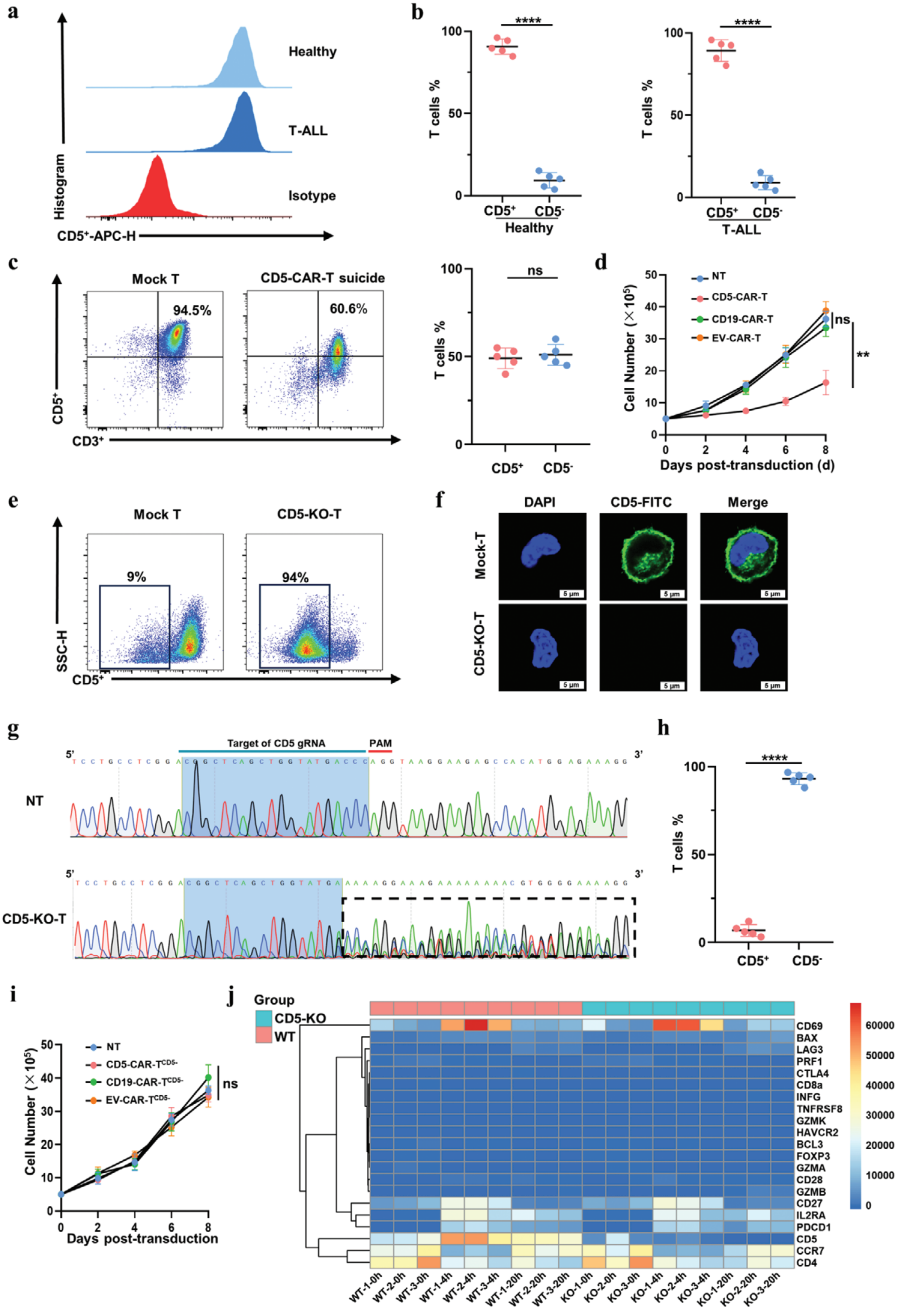
Effective disruption of CD5 gene in T cells with CRISPR/Cas9 system prevents fratricide without compromising T cell function. a, b) Flow cytometric analysis of surface expression of CD3 and CD5 in bulk peripheral blood mononuclear cells from healthy donors: CD3^+^CD5^+^ (mean, 90.8% ± 6.0%) versus CD3^+^CD5^−^(mean, 9.5% ± 5.7%) (*n* = 5, *p *< 0.0001, T‐test) and T‐ALL patients: CD3^+^CD5^+^ (mean, 87.95% ± 7.85%) and CD3^+^CD5^−^ (mean, 9.75% ± 5.55%) (*n* = 5, *p* < 0001, T‐test). c) Flow cytometric analysis of surface expression of CD3 and CD5 in CD5‐CAR‐T cells after fratricide: CD3^+^CD5^+^ (mean, 40.75% ± 7.5%) and CD3^+^CD5^−^ (mean, 53.5% ± 13.5%) (*n* = 5, *p* = ns, T‐test). d) Expansion kinetics and viability of CD5‐CAR‐T cells. CD5‐CAR‐T cells had decreased viability compared with CD19‐CAR‐T and NT‐T cells (*n* = 3, *p* < 0.01, 2‐way ANOVA). e) Flow cytometric analysis of T cells before and after CD5 depletion. f) Immunofluorescence for verifying depletion of CD5 by CLSM. The scale bar represents 5 µm. g) Sequencing analysis of the CD5 gene loss. h) Flow cytometric analysis of CD5‐KO efficacy of T cells: CD3^+^CD5^+^ (mean, 7.6% ± 4.4%) versus CD3^+^CD5^−^ (mean, 92.4% ± 4.4%) (*n* = 5, *p* < 0.0001, T‐test). i) Expansion kinetics and viability of CD5‐CAR‐T^CD5−^ cells. CD5‐CAR‐T^CD5−^ cells had similar expansion to NT‐T cells (*n* = 3, *p* = ns, 2‐way ANOVA). j) RNA‐seq analysis of functional‐related genes in T cells. T‐test was used to compare the statistical difference between 2 groups, and one‐way or two‐way analysis of variance with Sidak's or Tukey's multiple comparison tests was used to compare 3 or more groups (^*^ *p* < 0.05, ^**^ *p* < 0.01, ^***^ *p* < 0.005, ^****^ *p* < 0.0001). ns, not significant.

The presence of CD5^+^ T cells in peripheral blood was analyzed by flow cytometry in both healthy donors and T‐ALL patients (Figure **2**a). CD5^+^ T cells were present in all samples, with frequencies ranging from 84.8% to 96.8% in healthy donors and 80.1% to 95.8% in T‐ALL patients (Figure 2b). The expression of CD5‐27‐Nb on the T‐cell surface led to the fratricide of CD5‐CAR‐T cells, resulting in a decreased frequency of CD5^+^ T cells (Figure 2c) and a reduction in the CD5‐CAR‐T cell population (Figure 2d). To sustain the expansion and activity of CD5‐CAR‐T cells, the CD5 gene was disrupted in T cells using CRISPR/Cas9 prior to CAR transduction, eliminating CD5 expression on the T‐cell surface. CRISPR/Cas9 mediate CD5 gene disruption was performed before CAR transduction in this study, resulting in the loss of CD5 expression on the surface of T cells (Figure 2e,f). The guide RNA sequence used for targeting CD5 is listed in Table S1 (Supporting Information). Sequencing analysis confirmed the successful disruption of the CD5 gene in treated cells (Figure 2g). The frequency of CD5^+^ T cells was reduced to ≈9% (Figure 2h). Importantly, this genetic modification prevented fratricide following CAR transduction into CD5^−^ T cells (Figure 2i). Furthermore, analysis of the Gene Expression Omnibus database (GSE148821) revealed that the loss of the CD5 gene did not affect the expression of functional genes related to T‐cell activity (Figure 2j).

### 2.4 Generation of Fratricide‐Resistant γδT^CD5−^ Cells

**Figure 3:**
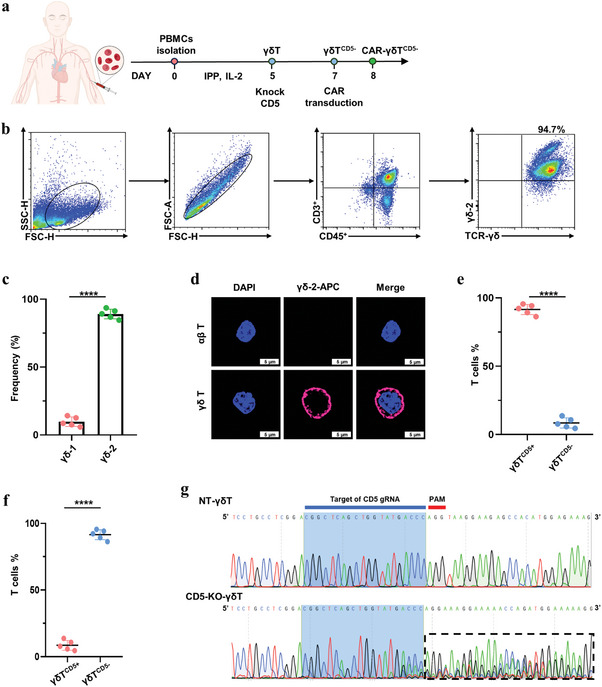
Generation of fratricide‐resistant γδT^CD5−^ cells. a) Schematic presentation of γδT cell enrichment. b) Gate strategy of γδT cells. c) Flow cytometric analysis of γδ−1 and γδ−2 expression in γδT cells stimulated from PBMCs: γδ−1 (mean, 10.05% ± 4.15%) versus γδ−2 (mean, 89.1% ± 4.5%) (*n* = 5, *p *< 0001, T‐test). d) Immunofluorescence for verifying γδ−2 expression in γδT cells by CLSM. Scale bar represents 5 µm. e) Flow cytometric analysis of surface expression of CD3 and CD5 in γδT cells. CD3^+^CD5^+^ (mean, 90.9% ± 4.6%) versus CD3^+^CD5^−^ (mean, 4.5% ± 4.6%) (*n* = 5, *p* < 0.0001, T‐test). f) Flow cytometric analysis CD5 expression of γδT cells before and after CD5 depletion. CD3^+^CD5^+^ (mean, 9.1% ± 4.6%) versus CD3^+^CD5^−^ (mean, 91.1% ± 4.8%) (*n* = 5, *p* < 0.0001, T‐test). g) Sequencing analysis of the CD5 gene loss in γδT cells. T‐test was used to compare the statistical difference between 2 groups (^*^ *p* < 0.05, ^**^ *p* < 0.01, ^***^ *p* < 0.005, ^****^ *p* < 0.0001).

A previous study indicated the percentage of γδT cells in the peripheral blood varies from 3% to 10%.^[^ ^30^ ^]^ γδT cells were enriched and expanded by stimulation of IPP and IL‐2 from PBMCs (**Figure** **3**a), resulting in γδ−2‐T cells reaching a concentration of ∼90% (Figure 3b,c). CLSM results further confirmed the successful stimulation of γδ−2‐T cells (Figure 3d). The frequency of γδT^CD5+^ cells in the samples was determined by flow cytometry, ranging from 84.6% to 93.4% in all samples. In this context, CRISPR/Cas9 was performed to knock out the CD5 gene of γδT cells before CAR transduction to prevent fratricide (Figure 3a). Flow cytometry and sequencing analysis were conducted to verify the effective loss of CD5 expression and gene (Figure 3f,g).

**Figure 4:**
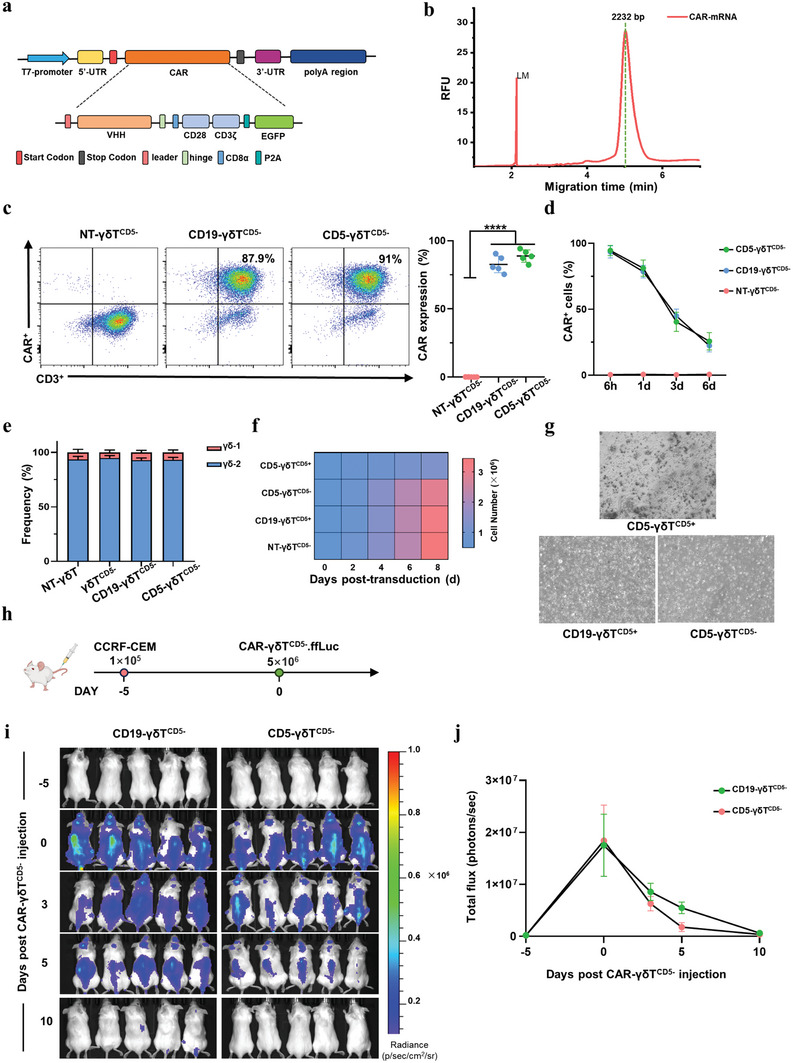
Fratricide‐resistant CD5‐CAR γδT^CD5−^ cells constructed by anti‐CD5‐CAR‐mRNA. a) Schematic representation of in vitro transcribed mRNA encoding anti‐CD5‐VHH CAR. b) Assessment of the integrity and length of IVT CAR‐mRNA. c) Representative flow cytometry analysis showing CD5‐CAR expression after transduction of anti‐CD5‐VHH CAR mRNA. (mean transduction efficiency 88.25% ± 5.95%, *n* = 5, *p* < 0.0001, T‐test). d) Evaluation of CAR expression of CD5‐CAR‐γδT^CD5−^ by flow cytometry analysis from 6 h to 6 days. e) Immunophenotype of CD5‐CAR‐γδT^CD5−^ cells post transduction. f) Expansion kinetics and viability of CD5‐CAR‐γδT^CD5−^ cells. g) Absence of fratricide observed in CD5‐CAR‐γδT^CD5−^ under the light microscope. h) Schematic of CD5‐CAR‐γδT^CD5−^ in vivo persistence experiment: NSG mice were injected with 1 × 10^5^ CCRF‐CEM cells 5 days prior to a single dose of 5 × 10^6^ CD5‐CAR‐γδT^CD5−^.ffLuc cells. i) Corresponding bioluminescence imaging. j) Quantitative bioluminescence data. T‐test was used to compare the statistical difference between 2 groups (^*^ *p* < 0.05, ^**^ *p* < 0.01, ^***^ *p* < 0.005, ^****^ *p* < 0.0001).

After the loss of CD5 gene in γδT cells, γδT^CD5−^ cells were transduced by mRNA electroporation to express CD5‐Nb‐CAR (CD5‐CAR‐γδT^CD5−^) or a control CD19‐Nb‐CAR (CD19‐CAR‐γδT^CD5−^) (**Figure** **4**a). Capillary electrophoresiswas used to ensure the integrity of IVT CAR‐mRNA (Figure 4b). The γδT^CD5−^ cells exhibited high CAR expression levels ranging from 75.4% to 94.2% (Figure 4c). The expression of CAR on γδT^CD5−^ cells was sustained for ≈1 week and gradually declined following their generation (Figure 4d). Besides, after CAR transduction by electroporation, the phenotype and expansion of γδT cells did not change significantly (Figure 4e,f). The percentage of dead cells ranged from 1.5% to 3.2% on day 2, 4.4% to 10.3% on day 5, 5.2% to 13.1% on day 8 after electroporation (Figure S2, Supporting Information). Due to the loss of the CD5 gene, the fratricide of CD5‐CAR‐γδT^CD5−^ was not observed (Figure 4f,g). To evaluate the endurance of CD5‐CAR‐γδT^CD5−^, a schematic of in vivo persistence experiment is shown in Figure 4g. To be more specific, NOD/SCID IL‐2RγCnull (NSG) mice were injected with 1 × 10^5^ CCRF‐CEM cells 5 days prior to a single dose of 5 × 10^6^ CD5‐CAR‐γδT^CD5−^ cells (Figure 4h). Prior to the injection of CAR‐T cells into NSG mice, the number of CCRF‐CEM cells in the mice's blood was evaluated to confirm successful engraftment (Figure S3, Supporting Information). Throughout the observation period, results from bioluminescence imaging and quantitative da = ta indicated that mRNA‐engineered CD5‐CAR‐γδT^CD5−^.ffLuc cells remained viable in vivo for ≈5 days before their numbers started to decline, primarily due to their antitumor activity against CCRF‐CEM cells. This finding suggests that subsequent administrations of CD5‐CAR‐γδT^CD5−^ cells might amplify the therapeutic benefits, highlighting the potential for repeated dosing to enhance treatment efficacy.

### 2.5 CD5‐CAR‐γδT^CD5−^ Demonstrates Specificity and Exhibits Potent Cytotoxicity Against Malignant T‐Cell Lines In Vitro

**Figure 5:**
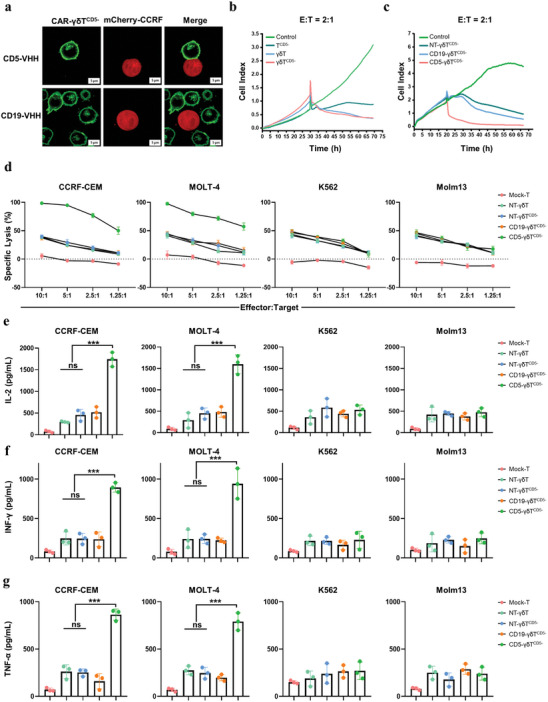
In vitro antitumor efficacy of CD5‐CAR γδT^CD5−^ cells. a) Immunofluorescence for verifying the specific binding ability of CD5‐CAR γδT^CD5−^ cells (green) with CCRF‐CEM‐mCherry (red) by CLSM. Scale bar represents 5 µm. b) Detection of the ability of T^CD5−^ cells, γδT cells, and γδT^CD5−^ cells to lyse target cells (CD5‐HeLa) using RTCA. c) Detection of the ability of CD5‐CAR‐γδT^CD5−^ cells, CD19‐CAR‐γδT^CD5−^ cells, NT‐γδT^CD5−^ cells to lyse target cells (CD5‐HeLa) using RTCA. d) CD5‐CAR‐γδT^CD5−^ specifically killed CCRF‐CEM and MOLT‐4 cells under different E:T ratios. (e‐g) CD5‐CAR‐γδT^CD5−^ cells, CD19‐CAR‐γδT^CD5−^ cells, NT‐γδT^CD5−^ cells, and NT‐γδT cells were co‐cultured with media, Molm13, K562, CCRF, or MOLT‐4 at a 2:1 E:T ratio. Supernatants were harvested after 24 h and analyzed for IFN‐γ, IL‐2, and TNF‐α by ELISA (*n* = 3, 1‐way ANOVA, ^***^ *p* < 0.005). ns, not significant.

The cytotoxicity of CAR‐γδT cells was directly evaluated through a co‐culture experiment involving effector CAR‐γδT cells and malignant T‐cell lines. Fluorescence microscopy visualization revealed that, at lower effector‐to‐target (E:T) ratios, CD5‐CAR‐γδT^CD5−^ cells exhibit superior recognition capabilities for CCRF‐CEM cells compared to CD19‐CAR‐γδT^CD5−^ cells (**Figure** **5**a). Subsequent to the introduction of CD5‐HeLa cells into the E‐Plate, the real‐time dynamic alterations of the co‐cultured cells were monitored using the RTCA system. γδT^CD5−^ cells showed a more robust ability to lyse CD5‐HeLa than T^CD5−^ cells, although not entirely effective (Figure 5b). Compared to CD19‐CAR‐γδT^CD5−^ and non‐transduced (NT)‐γδT^CD5−^, CD5‐CAR‐γδT^CD5−^ cells exhibited a more efficient ability to lyse CD5‐HeLa cells (Figure 5c).

To further confirm this, stable luciferase‐expression cell lines were generated from parental CCRF‐CEM, MOLT‐4, K562, and Molm13 cell lines to perform luciferase‐based determination of cytotoxicity of CAR‐γδT. CD5‐CAR‐γδT^CD5−^ cells efficiently lysed CD5^+^ CCRF‐CEM and MOLT‐4 cells but not CD5^−^ K562 and Molm13 cells, demonstrating their specific recognition of CD5. NT‐γδT cells were used as a control to evaluate unspecific lysis. The NT control cells showed weak cytotoxicity against all 4 cell lines but no selectivity to CD5 expression. Similar results were observed at different ratios of E:T (Figure 5d). Besides, mRNA‐CD5‐CAR‐γδT^CD5−^ cells show a similar antitumor efficacy with the CD5‐CAR‐γδT^CD5−^ cells constructed by lentivirus (LV‐CD5‐CAR‐γδT^CD5−^) (FigureS4, Supporting Information). These data indicate that the engineered CD5‐CAR‐γδT^CD5−^ cells specifically lysed CCRF‐CEM and MOLT‐4 cells in vitro.

To assess the effector function of CD5‐CAR‐γδT^CD5−^ cells, cytokines were measured during the in vitro cytotoxicity assay. In comparison with NT control cells, CD5‐CAR‐γδT^CD5−^ cells produced a broad range of cytokines when co‐cultured with CD5^+^ target cells. Increased expression of effector cytokines such as interleukin‐2 (IL‐2), interferon‐γ (IFN‐γ), and tumor necrosis factor‐α (TNF‐α) when co‐cultured with CD5^+^ target cells, while CD5‐CAR‐γδT^CD5−^ cells produced few or no cytokines when co‐cultured with CD5^−^ target cells (Figure 5e–g). Besides, the detections of cytokine secretion of interleukin‐10 (IL‐10) and transforming growth factor‐β (TGF‐β) were performed to elucidate the inhibitory role of CD5‐CAR‐γδT^CD5−^. As depicted in FigureS5 (Supporting Information), low expression of IL‐10 and TGF‐β was observed when CD5‐CAR‐γδT^CD5−^ cells were co‐cultured with target cells, suggesting a focused and potent immunotherapeutic action against CD5^+^ malignancies.

### 2.6 CD5‐CAR‐γδT^CD5‐^ Demonstrates Antitumor Efficacy Against Malignant T Cell Lines In Vivo

**Figure 6:**
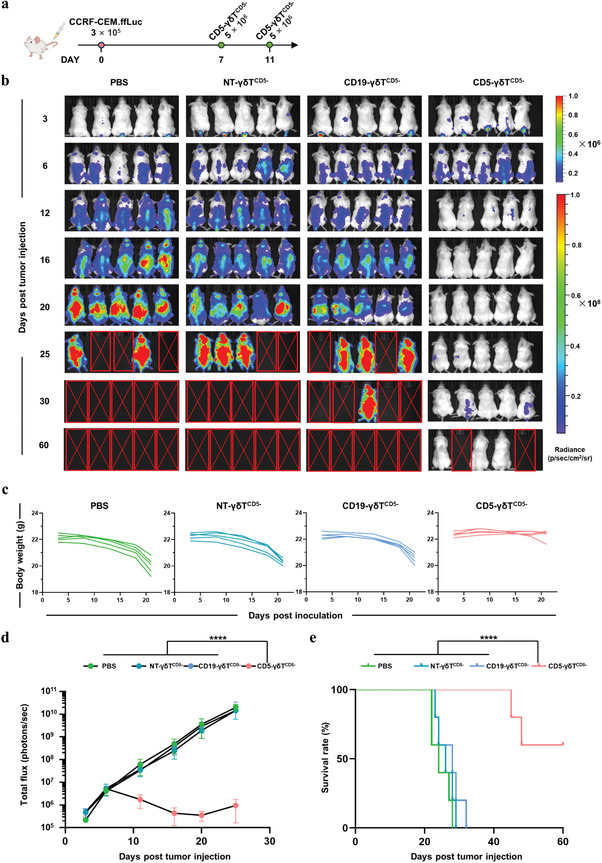
Antitumor activity of CD5‐CAR‐γδT^CD5−^ cells in CCRF‐CEM xenograft model. a) Schematic representation of CD5‐CAR‐γδT^CD5−^ in vivo treatment for CCRF‐CEM.ffLuc xenograft model: NSG mice were intravenously injected with 3 × 10^5^ CCRF‐CEM.ffLuc cells on day 0 and double doses of 5 × 10^6^ CD5‐CAR‐γδT^CD5−^ cells on day 7 and 11, respectively. b) Corresponding bioluminescence imaging (*n* = 5). c) Body weight curve. d) Quantitative bioluminescence data (*n* = 5, 2‐way ANOVA). Mice treated with CD5‐CAR‐γδT^CD5−^ cells exhibited significantly decreased bioluminescence (^****^ *p* < 0.0001) compared to the CD5‐CAR‐γδT^CD5−^, NT‐γδT^CD5−^ and PBS‐treated groups. e) Survival curve (^****^ *p* < 0.0001, Mantel‐Cox log‐rank test). Mice treated with CD5‐CAR‐γδT^CD5−^ cells showed significantly increased survival (*p *< 0.0001) compared to CD19‐CAR‐γδT^CD5−^, NT‐γδT^CD5−^ and PBS‐treated groups.

After confirming the specificity and reactivity of CD5‐CAR‐γδT cells against human T‐ALL in vitro, their antitumor activity in vivo was further assessed. We first developed a xenogeneic model of systemic T‐ALL using the malignant T cell lines engineered to express the firefly luciferase reporter gene (CCRF‐CEM.ffLuc and MOLT‐4.ffLuc). NSG mice were intravenously injected with 5  ×  10^5^ CCRF‐CEM.ffLuc cells on day 0. T‐ALL progression was observed on day 3 after inoculation. On days 7 and 11 after tumor inoculation, mice were treated with intravenous injection of either 5  ×  10^6^ CD5‐CAR‐γδT^CD5−^, CD19‐γδT^CD5−^, NT‐γδT^CD5−^ cells, or phosphate‐buffered saline (PBS) as a control (**Figure** **6**a). Notably, the CD5‐CAR‐γδT^CD5−^ treated group demonstrated rapid clearance of CCRF‐CEM.ffLuc cells, achieving and sustaining remission more effectively than both the CD19‐γδT^CD5−^ and NT‐γδT^CD5−^ treated groups (Figure 6b,e). In contrast to the mice treated with CD19‐γδT^CD5−^ or NT‐γδT^CD5−^ cells, which exhibited a massive leukemic burden by bioluminescence imaging, the mice treated with CD5‐CAR‐γδT^CD5−^ cells were practically tumor cells free by day 16, although tumor recurrence was observed on day 25 (Figure 6b). Impressively, mice treated with CD5‐CAR‐γδT^CD5−^ significantly delayed tumor progression and prolonged the survival time of tumor‐bearing mice (Figure 6b,d). Following the suspension of administration, 3 out of 5 mice remained free from tumor recurrence up to day 60 post‐treatment (Figure 6b,e). The body weight of tumor‐bearing mice treated with CD5‐CAR‐γδT^CD5‐^ cells showed no significant difference, indicating a tolerable safety profile (Figure 6c). In stark contrast, control mice treated with CD19‐γδT^CD5−^, NT‐γδT^CD5−^, or PBS showed a rapid progression of T‐ALL, with most of them dying around day 30 (Figure 6b,e).

**Figure 7:**
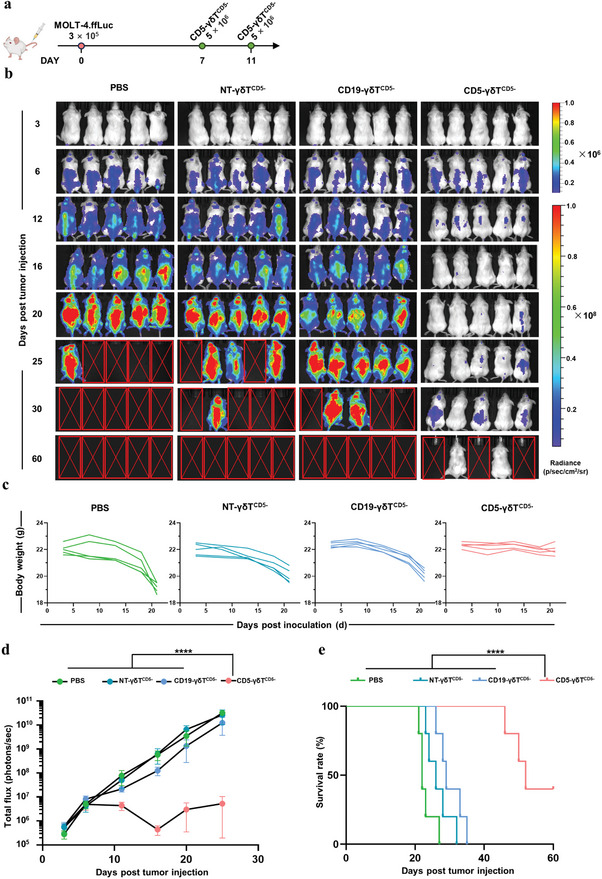
Antitumor activity of CD5‐CAR‐γδT^CD5−^ cells in MOLT‐4 xenograft model. a) Schematic of CD5‐CAR‐γδT^CD5−^ in vivo treatment of MOLT‐4 xenograft model: NSG mice were intravenously injected with 3 × 10^5^ MOLT‐4.ffLuc cells on day 0 and double doses of 5 × 10^6^ CD5‐CAR‐γδT^CD5−^ cells on day 7 and 11, respectively. b) Corresponding bioluminescence imaging (*n* = 5). c) Body weight curve. d) Quantitative bioluminescence data (*n* = 5, 2‐way ANOVA). Mice treated with CD5‐CAR‐γδT^CD5−^ cells exhibited significantly decreased bioluminescence (^****^ *p* < 0.0001) compared to CD5‐CAR‐γδT^CD5−^, NT‐γδT^CD5−^ and PBS‐treated groups. e) Survival curve (^****^ *p* < 0.0001, Mantel‐Cox log‐rank test). Mice treated with CD5‐CAR‐γδT^CD5−^ cells showed significantly increased survival (*p* < 0.0001) compared to CD5‐CAR‐γδT^CD5−^, NT‐γδT^CD5−^ and PBS‐treated groups.

Subsequently, to further validate the antitumor effect of CD5‐CAR‐γδT^CD5−^ in vivo, we selected MOLT‐4.ffLuc cells to establish another T‐ALL mouse tumor model (**Figure** **7**a). Similar antitumor efficacy of CD5‐CAR‐γδT^CD5−^ was verified in MOLT‐4.ffLuc cells xenogeneic model. Remarkably, the CD5‐CAR‐γδT^CD5−^ treatment facilitated an expedited clearance of MOLT‐4.ffLuc cells and ensured an extended period of remission in comparison to both CD19‐γδT^CD5−^ and NT‐γδT^CD5−^ treated groups (Figure 7b,e). Unlike mice treated with CD19‐γδT^CD5−^ or NT‐γδT^CD5−^ cells, which exhibited a massive leukemic burden by bioluminescence imaging, the mice treated with CD5‐CAR‐γδT^CD5−^ cells were practically tumor cells free by day 16, although tumor recurrence was observed on day 20 (Figure 7b,d). After the suspension of administration, 2 out of 5 mice remained free from tumor resurgence up to day 60 post‐treatment (Figure 7b,e). No significant body weight loss of tumor‐bearing mice treated with CD5‐CAR‐γδT^CD5−^ cells were observed (Figure 7c), suggesting the treatment was well tolerated. However, the discontinuation of CD5‐CAR‐γδT^CD5−^ administration post‐day 11 led to a lack of sustained efficacy, resulting in tumor relapse in some instances across both CCRF‐CEM and MOLT‐4 xenograft models. This outcome highlights the necessity for ongoing treatment to prevent tumor recurrence and underscores the importance of further research to optimize dosing schedules for prolonged antitumor effects.

Preliminary data from the CD5‐CAR‐T clinical trial treatment revealed a good safety profile, but efficacy was modest and non‐persistent, likely due to CAR‐T cell fratricide.^[^ ^31^ ^]^ Previous studies have demonstrated that the loss of the CD5 gene does not compromise T cell function but enhances antitumor efficacy by promoting activation and proliferation.^[^ ^14^, ^32^ ^]^ Therefore, to boost persistence and prevent fratricide of CD5‐CAR‐T cells, CRISPR/Cas9 was employed to knock out the CD5 gene before constructing γδT cells in our study.

While CAR‐T therapy has received regulatory approval, ongoing research is expanding its application to solid tumors and other hematological malignancies. However, the FDA (The Food and Drug Administration) has raised concerns regarding the potential for T‐cell malignancies following BCMA‐directed or CD19‐directed autologous CAR‐T cell immunotherapies. In its statement, the FDA is explicit that, with all gene therapy products using integrating vectors (lentiviral or retroviral vectors), the potential risk of developing secondary malignancies is categorized as a class warning. As mRNA is not integrated into the host genome, there is no risk of transgene‐mediated mutation and no unidentified long‐term risk. In this case, mRNA‐engineered CAR‐T therapy efficiently avoids the risk of developing secondary malignancies. In clinical trials, CAR‐mRNA delivery into T cells was achieved through electroporation.^[^ ^33^ ^]^ Therefore, our study constructed mRNA‐CAR‐γδT to evaluate its antitumor efficacy in vitro and in vivo. In this context, IVT was performed to produce CD5‐VHH‐directed CAR‐encoding mRNA, and electroporation generated mRNA‐based CAR‐γδT cells with comparable CAR expression after 24 h.

Despite the promising potential of mRNA‐based CAR‐T therapies, certain challenges exist. From a transport perspective, negatively charged mRNA lacks the inherent ability to diffuse into T cells. To address this limitation, techniques such as mRNA electroporation and the use of mRNA delivery carriers, such as lipid nanoparticles (LNPs), have been employed to facilitate mRNA internalization and trafficking.^[^ ^34^ ^]^ Recent studies suggest that LNPs outperform electroporation, highlighting their potential for mRNA‐LNP delivery in ex vivo CAR‐T cell modification.^[^ ^35^ ^]^ Therefore, the development of biomaterials for efficient CAR transduction in vitro or in vivo, replacing electroporation, becomes crucial. Regarding the persistence of mRNA‐CAR‐T, compared to mRNA vaccines, CAR therapy demands higher mRNA doses and repeated administration to achieve long‐lasting therapeutic effects. To overcome this challenge, ongoing efforts are needed to explore new technologies such as sustained‐release mRNA delivery systems, mRNA sequence optimization, sa/ta RNA, and circ RNA to reduce the dose and extend the duration of CAR expression.

## 3 Conclusion

The VHH library was successfully constructed and the CD5‐27 Nb was selected as the optimal antigen‐targeting domain for CAR construct. γδT cells were amplified from PBMCs of healthy donors through IPP and IL‐2 stimulation. To prevent fratricide, CRISPR/Cas9 was performed to knock out the CD5 gene of γδT (γδT^CD5−^). To mitigate the risks associated with viral vector integration into the cell genome, which potentially induces tumor formation, CAR mRNA was transcribed in vitro to construct CD5‐CAR‐γδT^CD5−^. This approach ensures safety, rapidity, and cost‐effectiveness in the construction of CD5‐CAR‐γδT^CD5−^. Remarkably, CD5‐CAR‐γδT^CD5−^ cells exhibited efficient antitumor efficacy against malignant T‐cell lines both in vitro and in vivo. This significant achievement underscores the potential of CD5‐CAR‐γδT^CD5−^ therapy as a promising avenue for future clinical applications in the treatment of T‐cell acute lymphoblastic leukemia. Our findings highlight the therapeutic potential of leveraging specific antigen‐targeting domains combined with the innovative use of CRISPR/Cas9 gene editing and mRNA technology to create more effective and safer CAR‐γδT cell therapies.

## 4 Experimental Section

### Cells and Mice

HEK 293T, HeLa, CCRF‐CEM, MOLT‐4, Molm13, and K562 were purchased from the American Type Culture Collection (ATCC) and cultured in DMEM (Gibco) or RIPM‐1640 (Gibco). γδT^CD5−^ was generated by knockout of CD5 gene using CRISPR/Cas9 RNP. 10% fetal calf serum and 1.0 mmol L^−1^ of the penicillin‐streptomycin combination were utilized in all mediums (Hyclone). Cell lines were grown at 37 °C in a humid incubator with 5% CO2. Female NSG mice aged 6–8 weeks were used in this study. They were obtained from the Model Animal Resource Information Platform of Nanjing University (Nanjing, P. R. China) and housed in the animal vivarium of the State Key Laboratory of Biotherapy at Sichuan University in a pathogen‐free environment. All mouse studies have followed a protocol approved by the Institutional Animal Care and Use Committee of Sichuan University. Ethical approval was given by the West China Hospital of Sichuan University Laboratory Animal Ethics Committee (approval number 2018212A). Patients/participants provided written informed consent to participate in this study (full name: Ethics Committee of West China Hospital, Sichuan University) (reference numbers: 2018–061).

### γδT Cell Enrichment

Human peripheral blood mononuclear cells (PBMCs) were obtained from healthy donors. PBMCs were stimulated by IPP (500IU mL^−1^) and IL‐2 (500IU mL^−1^) for 3 days. Then, γδT cells were subsequently expanded with IL2. Cells were cultured for 7 to 12 days prior to being used for in vitro or in vivo experiments.

### CAR mRNA Synthesis

mRNA was produced using standard in vitro transcription methods, as previously described.^[^ ^36^ ^]^ Briefly, plasmid DNA encoding a second‐generation lentiviral vector for CD5 targeting VHH‐CAR bearing the CD3ζ and 4−1BB costimulatory domains was linearized overnight, followed by the production of mRNA using the T7 high‐yield RNA transcription kit (Vazyme Biotech Co., Ltd) as per manufacturer instructions. mRNA was then polyA‐tailed, capped, and purified using the RNeasy mini kit (Qiagen).

### CAR Construction Through Electroporation

γδT cells were collected and subsequently washed 3 times with MaxCyte buffer (HyClone) before being resuspended in MaxCyte buffer at a concentration of 1 × 10^7^ cells per 100 µl in an OC‐100 cuvette. Next, 100 µg of CAR mRNA was introduced into 1 × 10^7^ γδT cells through electroporation using the MaxCyte GTx system under the expanded T cells‐3 condition. The electroporated cells were transferred in prewarmed T cell expansion medium with IL‐2 and incubated at 37°C and 5% CO2 until further analyses were performed.

### Flow Cytometry

Anti‐human‐CD5, Anti‐human‐CD3, Anti‐human‐CD45, Anti‐human‐TCR‐γδ, Anti‐human‐γδ−2 antibody were purchased from Biolegend. Following 2  washes with PBS, the cells were incubated in a 1% BSA solution containing Fc blocker (Biolegend) for 30 min. The Zombie UV Fixable Viability Kit (BioLegend) was used to identify dead cells. CAR‐T cells were detected using GFP as a marker.

### Lactate Dehydrogenase (LDH) Assay

The LDH assay can be used to evaluate the antigen‐specific cytotoxicity of CD5‐CAR‐γδT^CD5−^ cells in an in vitro model at E:T ratios of 1.25:1, 2.5:1, 5:1, and 10:1. The LDH cytotoxicity test detection kit (Beyotime, China) was used in accordance with the manufacturer's instructions to determine the amount of LDH released into the cell culture supernatant.

### Real‐time Cytotoxicity Assays (RTCA)

Real‐time cytotoxicity assays (ACEA Bioscience, Inc. xCELLigence RTCA SP) were used to detect the ability of CAR‐T cells to lyse target cells. E‐plate 96 (ACEA Bioscience) was used to culture CD5‐HeLa tumor cells (10^4^ cells per well) for ≈24 h before the plates were treated with T cells (E:T = 2). Data were gathered and examined in line with the manufacturer‐specified methods (ACEA Bioscience, Inc. RTCA Software 2.1).

### Cytokine Production Assays

In a 24‐well plate at 37 °C without the use of exogenous cytokines, tumor cells were co‐cultured with 4 × 10^5^ reactive cells. The cytokines IL‐2, IFN‐γ, TNF‐α, IL‐10, and TGF‐β were assayed in the culture medium at 24 h later. Cytokines were measured using ELISA kits (BioLegend).

### IVIS

The mice were imaged with the IVIS using luminescent imaging by injecting intraperitoneally (i.p.) 150 mg kg^−1^ of body weight D‐luciferin. ROI measurements were taken through the Living Image 4.5 software.

### Graphs

Graphs were performed using GraphPad Prism Software version 8.3.0. Some of these graphs were obtained and modified from BioRender.com.

### Statistical Analysis

All results were expressed as means ± SEM (*n* = 3–5) as indicated. T‐test was used to compare the statistical difference between 2 groups, and one‐way or two‐way analysis of variance with Sidak's or Tukey's multiple comparison tests was used to compare 3 or more groups (*p < 0.05, **p < 0.01, ***p < 0.005, ****p < 0.0001). Survival rates were analyzed using the log‐rank (Mantel‐Cox) test. GraphPad Prism V.8.0.2 software was used to calculate the significance between the samples. FlowJo V.10.8.1 was used to analyze data of flow cytometry. All experiments were repeated, and representative figures were presented unless otherwise noted. *p* values ≤ 0.05 were considered significant.

Figure 1a, Figure 3a, Figure 4h, Figure 6a, and Figure 7a and ToC figure are created with BioRender.com.

## Conflict of Interest

The authors declare no conflict of interest.

## Supporting information

Supporting Information

## Data Availability

The data that support the findings of this study are available from the corresponding author upon reasonable request.
