# Supplementary material for: mRNA‐Engineered CD5‐CAR‐γδTCD5‐ Cells for the Immunotherapy of T‐Cell Acute Lymphoblastic Leukemia
Source: Adv Sci (Weinh). 2024 Jul 16;11(35):2400024. doi: 10.1002/advs.202400024 (PMC11425277; doi:10.1002/advs.202400024)
Supplement: Supplementary file 1 — Supporting Information [file ADVS-11-2400024-s001.docx]

**Supporting Information for mRNA-Engineered CD5-CAR-γδT^CD5-^ Cells for the Immunotherapy of T Cell Acute Lymphoblastic Leukemia**

**Table S1. CD5 guide RNA sequence in the study**

| Name | Sequence |
| --- | --- |
| CD5 gRNA | CGGCTCAGCTGGTATGACCC |


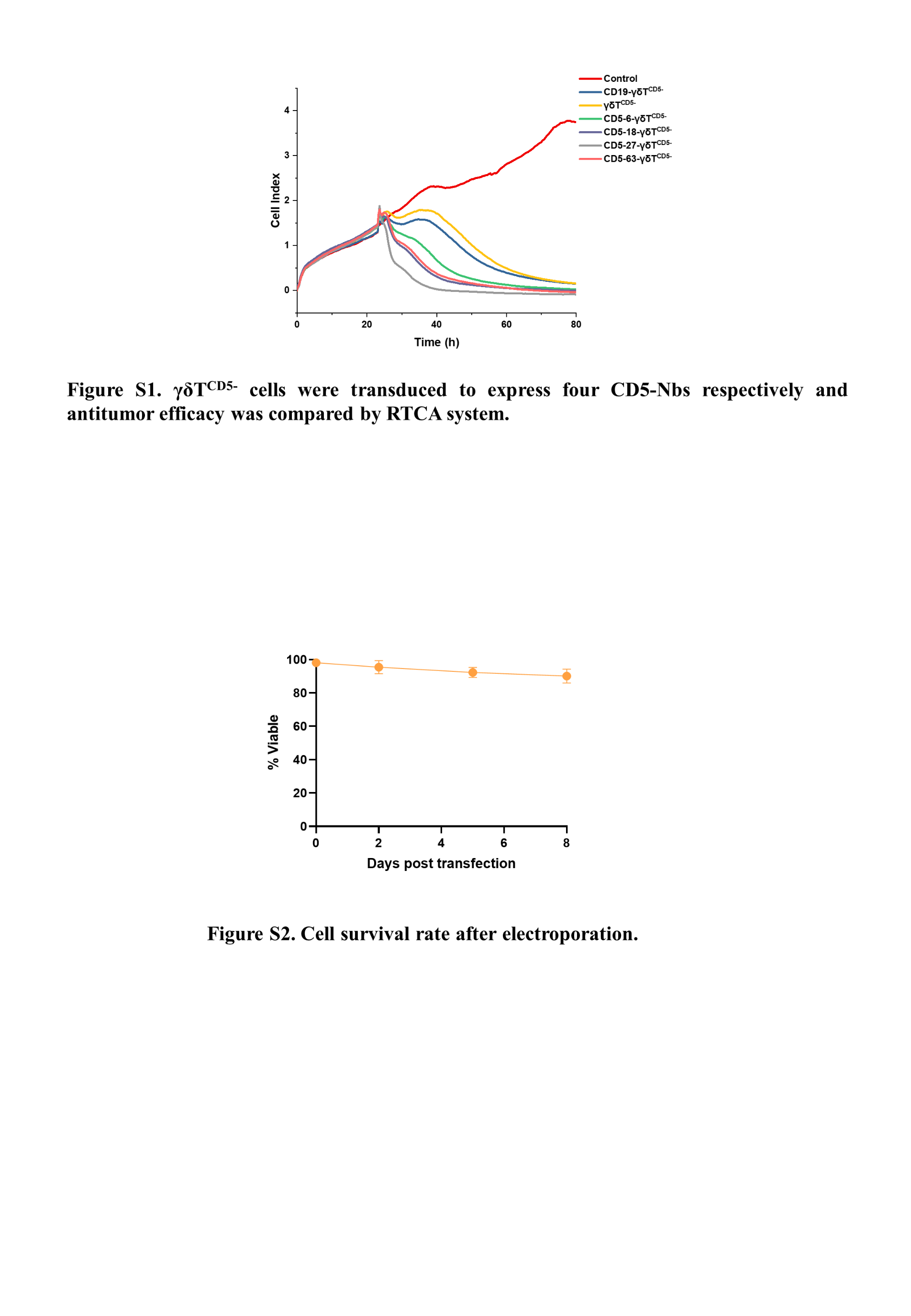


**Figure S1. γδT^CD5-^ cells were transduced to express four CD5-Nbs respectively and antitumor efficacy was compared by RTCA system.**


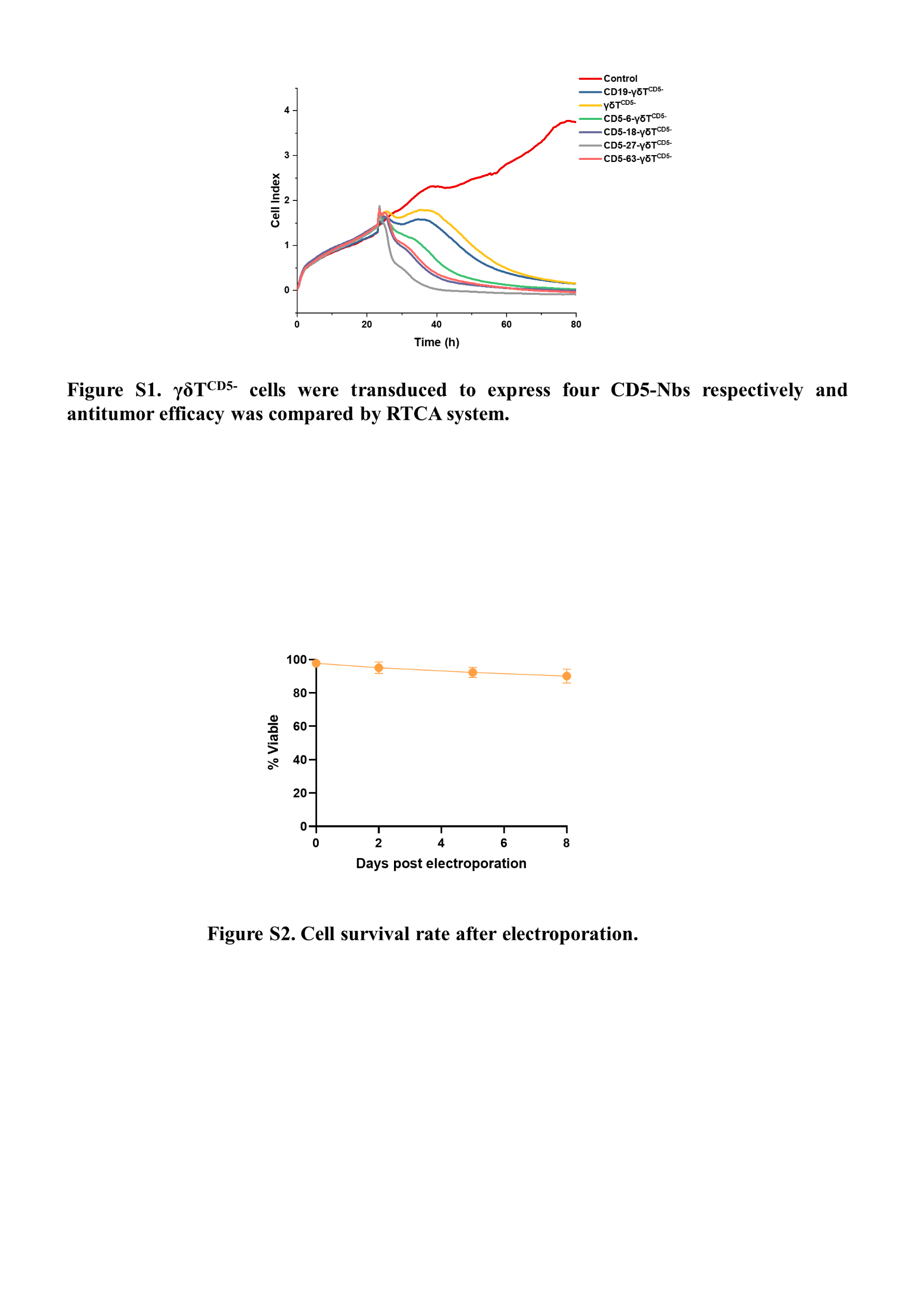


**Figure S2. Cell survival rate after electroporation.**


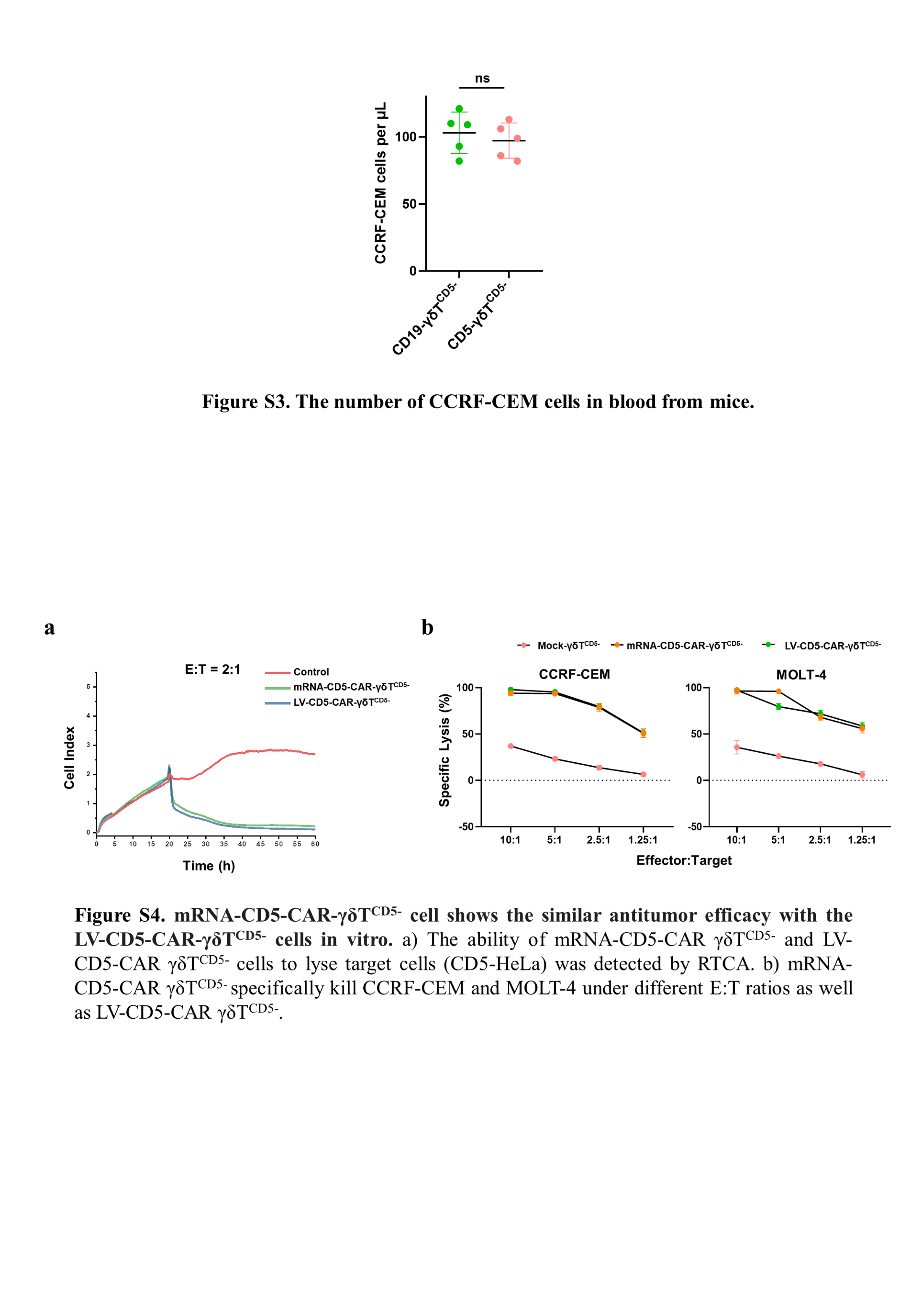


**Figure S3. The number of CCRF-CEM cells in blood from mice.**


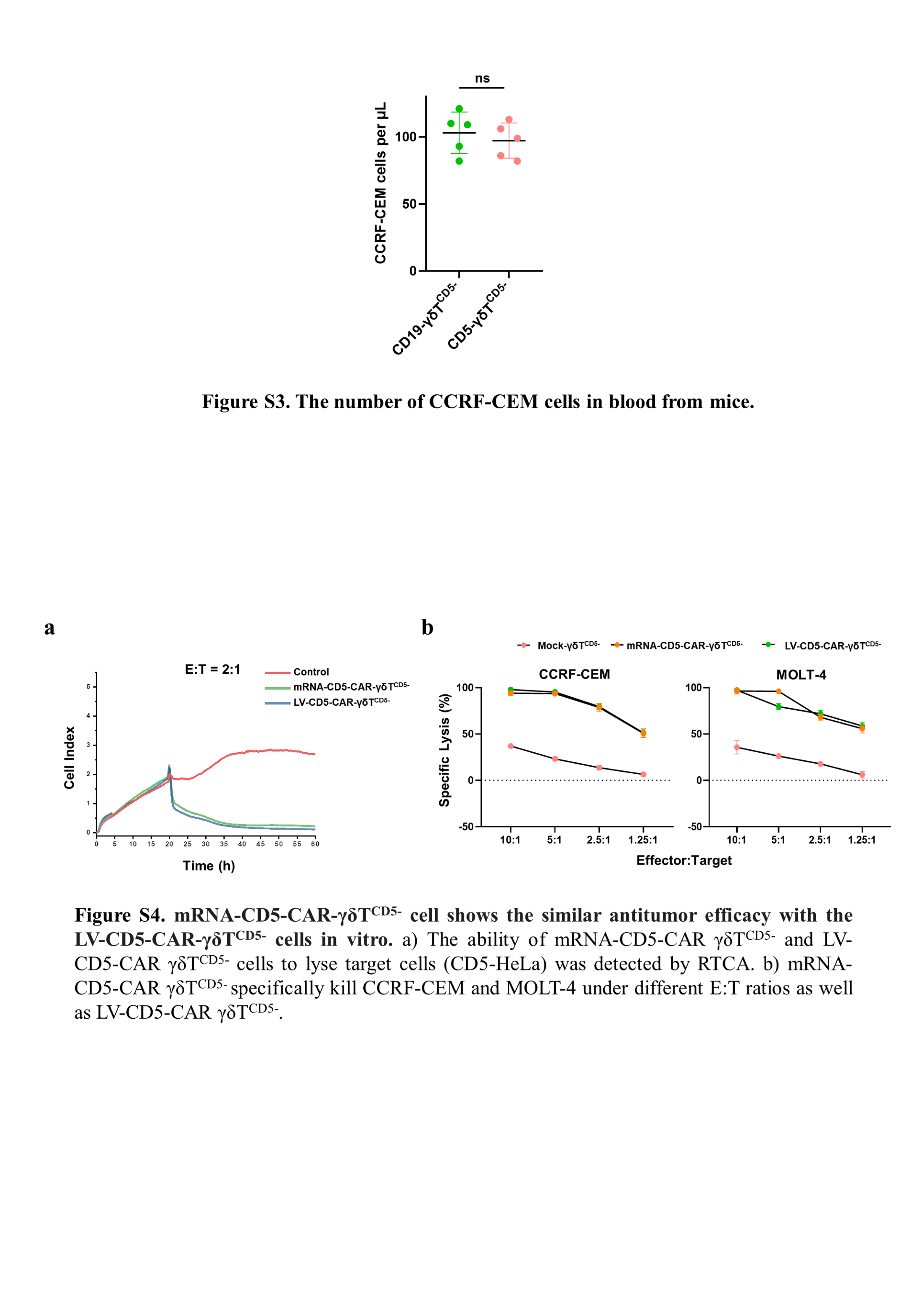


**Figure S4. mRNA-CD5-CAR-γδT^CD5-^ cell shows the similar antitumor efficacy with the LV-CD5-CAR-γδT^CD5-^ cells in vitro.** a) The ability of mRNA-CD5-CAR γδT^CD5-^ and LV-CD5-CAR γδT^CD5-^ cells to lyse target cells (CD5-HeLa) was detected by RTCA. b) mRNA-CD5-CAR γδT^CD5-^ specifically kill CCRF-CEM and MOLT-4 under different E:T ratios as well as LV-CD5-CAR γδT^CD5-^.

**
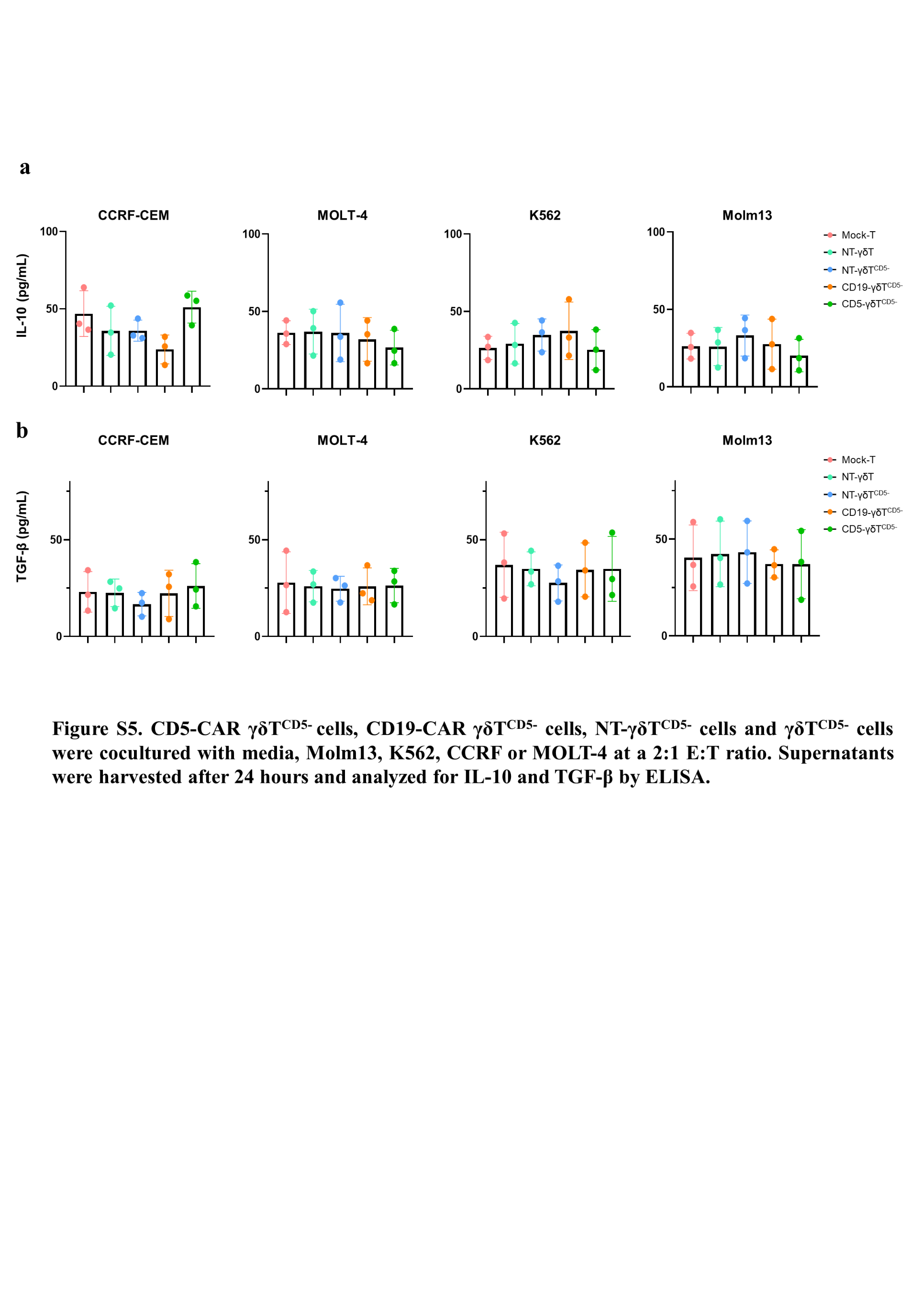
**

**Figure S5. CD5-γδT^CD5-^ cells, CD19-γδT^CD5-^ cells, NT-γδT^CD5-^ cells and NT-γδT cells were cocultured with media, Molm13, K562, CCRF or MOLT-4 at a 2:1 E:T ratio. Supernatants were harvested after 24 hours and analyzed for IL-10 and TGF-β by ELISA.**
